# Comparison of Influenza Epidemiological and Virological Characteristics between Outpatients and Inpatients in Zhejiang Province, China, March 2011–June 2015

**DOI:** 10.3390/ijerph14020217

**Published:** 2017-02-22

**Authors:** Wei Cheng, Zhao Yu, Shelan Liu, Xueying Zhang, Xiaoxiao Wang, Jian Cai, Feng Ling, Enfu Chen

**Affiliations:** 1 Zhejiang Provincial Centre for Disease Control and Prevention, Hangzhou 310051, China; wcheng@cdc.zj.cn (W.C.); zhy@cdc.zj.cn (Z.Y.); slliu@cdc.zj.cn (S.L.); xxwang@cdc.zj.cn (X.W.); jcai@cdc.zj.cn (J.C.); fengl@cdc.zj.cn (F.L.); 2 Field Epidemiology Training Programme of Zhejiang Province, Hangzhou 310051, China; 3 The University of Texas Health Science Center at Houston, School of Public Health, Houston, TX 77030, USA; xueying.zhang@uth.tmc.edu

**Keywords:** influenza surveillance, comparison, epidemic characteristics

## Abstract

Given the rapid rate of global spread and consequently healthcare costs related to influenza, surveillance plays an important role in monitoring the emerging pandemics in China. However, the characteristics of influenza in Southeast of China haven’t been fully studied. Our study use the surveillance data collected from 16 sentinel hospitals across Zhejiang Province during March 2011 through June 2015, including the demographic information and respiratory specimens from influenza-like illness (ILI) patients and severe acute respiratory illness (SARI) patients. As analysis results, most SARI and ILI patients were in the age group of 0–4 years old (62.38% of ILI and 71.54% of SARI). The respiratory specimens have statistically significantly higher positive rate for influenza among ILI patients than that among SARI patients (*p <* 0.001). The comparison between ILI patients and SARI patients shows no statistically significantly difference in detecting influenza virus type and influenza A virus subtype. The SARI and ILI patients were found to be positively correlated for overall positive rate (r = 0.63, *p* < 0.001), the weekly percentage of A(H1N1)pdm09 (r = 0.51, *p* < 0.001), influenza B virus (r = 0.17, *p* = 0.013), and A/H3N2 (r = 0.43, *p* < 0.001) among all the positive numbers. Our study demonstrated that the activities of influenza virus, including its subtypes, had a similar temporal pattern between ILI and SARI cases.

## 1. Introduction

Influenza virus is estimated to cause 3 to 5 million cases of severe illness and 250,000 to 500,000 deaths each year, while 5%–10% of adults and 20%–30% of children are infected with the influenza virus worldwide [1]. In lower and middle-income countries, influenza could result in large economic burden encompassing direct costs to the health service and households, and indirect costs of productivity losses [2,3].

Vaccination is a cost-effective way to reduce the public health and economic impacts caused by influenza. Because of the antigenic shift and drift of the virus, the influenza vaccine composition needs regular updates. Currently, the selection of strains for the annual influenza vaccine are primarily based on the predominant strain of influenza virus detected in influenza-like illness (ILI) [4]. However, ILI only represents a proportion of acute respiratory infections with mild clinical manifestations.

Severe acute respiratory illness (SARI) surveillance is another type of surveillance for severe influenza-associated disease. It is recommended to be monitored with established surveillance systems with ILI according to the guidelines of the World Health Organization (WHO) [5]. In China, patients meeting with the definition of SARI are required to have inpatient observation and necessary treatment. Due to the emerging high healthcare cost and severe consequences of SARI, effective vaccination is needed to reduce the incidence of SARI caused by influenza [6,7,8]. Therefore, whether influenza epidemiological and virological characteristics among SARI patients were consistent to those of ILI patients is critical to the effectiveness of current vaccination plan. 

Zhejiang Province located in the Southeastern China, featuring by its blooming economy and high density of population. It has over than 55 million permanent residents, which are distributed in 11 metropolitan areas. Influenza surveillance in Zhejiang Province launched at the same time with national surveillance in the year of 2001, and expanded to 16 ILI and four SARI sentinel hospitals by the year of 2011. Both ILI and SARI are monitored in the surveillance system. Although studies describing influenza surveillance with both ILI and SARI are well documented in the northern and southern hemispheres [9,10,11,12,13,14,15,16,17,18,19], few studies have fully compared the influenza epidemiological and virological characteristics between ILI and SARI cases. In this study, we used four-year continuous surveillance data to compare the epidemic and virological characteristics of influenza virus between ILI cases and SARI cases in Zhejiang Province.

## 2. Materials and Methods

### 2.1. Flu Surveillance

**Figure 1:**
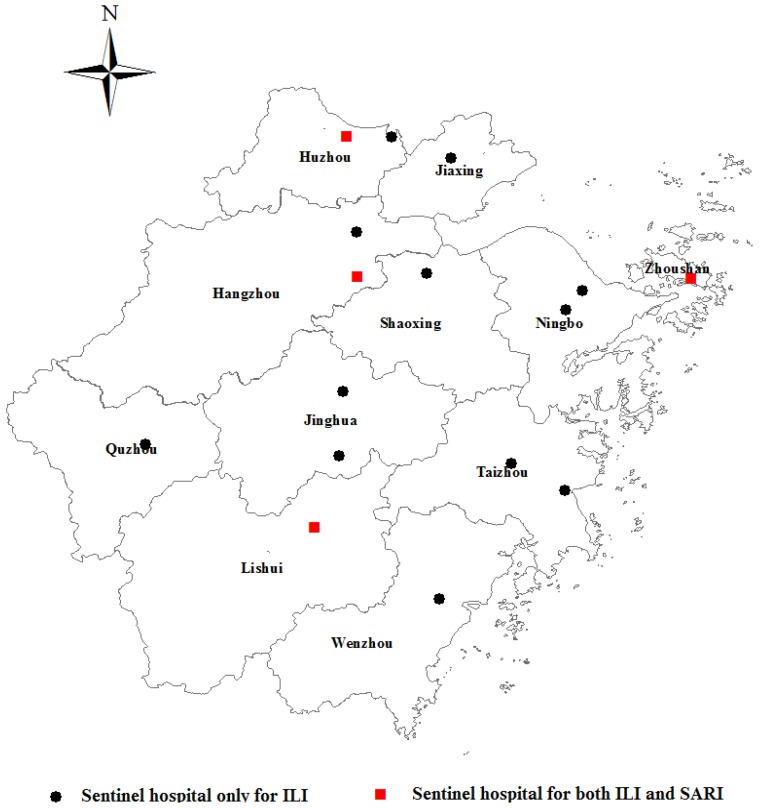
The geographical distribution of influenza sentinel hospitals in Zhejiang Province, China. (12 sentinel hospitals only for ILI, four sentinel hospitals for both ILI and SARI. ILI = Influenza-like illness; SAIR = severe acute respiratory illness).

The influenza surveillance network in Zhejiang Province is a part of the national influenza surveillance system [20]. The ILI surveillance in Zhejiang Province was initially launched in 2001. As to 2009, the ILI surveillance has been expanded into 16 sentinel hospitals that cover all of the 11 metropolitan areas in Zhejiang Province. The types of sentinel hospitals include general hospital, specialized clinic as well as maternal and children hospital. The SARI surveillance started in 2009. As to March 2011, the surveillance has expended to four sentinel hospitals including three general hospitals and one children’s hospital. All four SARI sentinel hospitals were selected from the existing ILI surveillance network (Figure 1).

### 2.2. Case Definition

ILI is defined as any person with sudden onset of fever >38 °C and cough or sore throat in the absence of other diagnosis [21]. The definition of SARI is varied by ages. A patient >5 years old is defined as having SARI if, upon or during admission, presenting an acute onset of elevated temperature (axillary temperature ≥38 °C) and cough or sore throat, as well as tachypnea (respiratory rate ≥25/min) or dyspnea (difficulty breathing). A patient ≤5 years old is defined as having SARI if, upon or during admission, presenting with acute onset of cough or dyspnea, and at least one of the following six signs or symptoms: (a) tachypnea(respiratory rate >60/min for ages <2 months, respiratory rate >50/min for ages 2 to <12 months, and respiratory rate >40/min for ages 1 to ≤5 years); (b) inability to drink or breastfeed; (c) vomiting; (d) convulsions; (e) lethargy or unconsciousness; (f) chest in-drawing or stridor in a calm child [4].

### 2.3. Data Collection

Each week, physicians from departments of pediatrics and respiratory, as well as emergency rooms in all of the 16 ILI sentinel hospitals were required to report the number of total visits to outpatient, as well as the number of outpatients who presented with non-specific symptoms that meet a case definition of ILI. As for four SARI sentinel hospitals, the number of SARI and total admissions from pediatrics ward, the respiratory medicine ward and the intensive care unit were also reported. All of the reported numbers in the above-mentioned departments were required to record by age classified as 0-, 5-, 15-, 25-, and 60- years old. Nasopharyngeal or throat swabs were required to collect from all of SARI cases and 5–15 ILI cases of each sentinel hospital on every week. The collection of swabs was conducted by trained nurses from who had not received antiviral drugs. In addition, a standardized case report form containing demographic and sample information was also required to complete among whose biological samples had been collected. Due to the outbreak of avian influenza A(H7N9) in April 2013, ILI surveillance was strengthened by increasing the weekly number of the biological samples collected in each hospital from 5–15 to 20. The biological samples were collected and saved in cryovial tubes, stored at 4 °C at the sentinel site, and then sent to regional center for disease control and prevention (CDC) within 48 h after the sample collected. The regional CDC laboratory tested influenza using real-time reverse transcription polymerase chain reaction (rRT-PCR) assay following the standard protocols. Specimens tested as positive for influenza A were further tested for subtypes (i.e., A(H1N1), A(H3N2), A(H1N1)pdm09, and A(H7N9)) using specific rRT-PCR. Once the laboratory complete, regional CDC submitted the laboratory results to the online surveillance system.

### 2.4. Statistical Methods

Data obtained from the surveillance system were reported weekly by the staff of the sentinel hospitals and laboratories. The mean and standard deviation or median and interquartile range (IQR) were calculated for continuous variables, and percentages were calculated for categorical variables. Chi-squared test and Fisher’s exact test were used to assess the differences of age, sex, season, influenza virus type/subtype, and influenza positive rate between all the sampled ILI and SARI cases. The weekly number of positive influenza by subtype and the percentage of specimens tested positively were plotted to describe seasonality and circulation of influenza types/subtypes among SARI and ILI cases. Spring, summer, autumn, and winter were defined from week 11 to 21, 22 to 38, 39 to 48, and 49 to 10 of the next year, respectively [22]. Cochran-Armitage trend test was used to analyze the trend change of influenza virus positivity with the increase of age. Spearman correlation was applied to analyze the linear relationship of the influenza virus positive rate, weekly percentage of influenza virus subtypes accounted for all the positive numbers between SARI and ILI patients. ILI percentage was calculated as the percentage of total outpatient visits that were due to ILI and SARI percentage was calculated as the percentage of total admissions that were due to SARI. To compare ILI percentage and SARI percentage of which can better reflect the activity of influenza virus among outpatients and inpatients, Spearman correlation analysis was used to assess the linear relationship between weekly ILI influenza-positive rate and ILI percentage, as well as weekly SARI percentage and SARI influenza-positive rate. A two-sided *p*-values were considered as statistically significant if it was found less than 0.05. All the statistics were conducted using SAS version 9.2 (SAS Institute, Cary, NC, USA).

### 2.5. Ethical Consideration

Verbal consent was obtained from all patients in prior to survey and specimen collection. For children aged under 15 years old, verbal consent was obtained from at least one parent or legal guardian. The influenza surveillance were a national-wide, governmental public health activity. Therefore, institutional review board approval was not required in China. In this study, the personal identifiers (e.g., names, address, occupations and so on) were not disclosed in order to maintain patient confidentiality, all the patient information was analyzed anonymously.

## 3. Results

### 3.1. Age Distribution among Total ILI and SARI Cases

**Figure 2:**
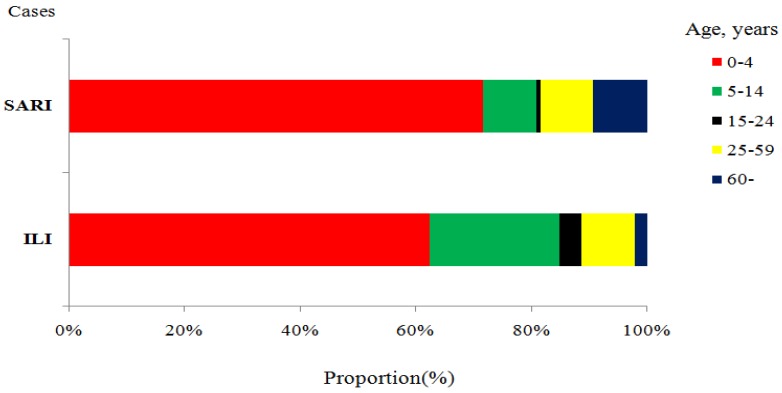
Age distribution among total reported ILI and SARI cases in Zhejiang Province, March 2011–June 2015 (ILI = Influenza-like illness; SAIR = severe acute respiratory illness).

From March 2011 through June 2015, a total of 944,370 ILI and 5425 SARI cases were reported to Zhejiang Province’s surveillance system. Age distributions shows that children aged 0–4 years old were the largest group of ILI (589,127, 62.38%) and SARI (3881, 71.54%) patients (Figure 2).

### 3.2. Demographic and Virologic Characteristics of the Sampled Patients

During the study period, 52,293 patients completed both the standardized case report and laboratory sample test, of which 46,868 (89.63%) were ILI patients and 5425 (10.37%) SARI patients. The median age of the tested patients was 15 years (IQR: 3–34), and the median age of ILI patients was significantly older than it of SARI patients (*p* < 0.001). In addition, the group of SARI patients had higher proportion of children at 0–4 years old (71.54% for SARI versus 27.82% for ILI), male (61.25% for SARI versus 50.67% for ILI), and patients enrolled in the winter (34.14% for SARI versus 28.32% for ILI) than those of ILI patients (Table 1).

### 3.3. Constitution of the Detected Influenza Virus and Influenza A Virus among ILI and SARI Patients

The most identified influenza virus were influenza A virus (61.97% among ILI, and 63.05% among SARI), followed by influenza B virus (37.92% among ILI, and 36.95% among SARI), and mixed type virus (0.11% among ILI; 0.00% among SARI), with no statistical significance between the two groups (*p* = 0.774). For influenza A virus in the ILI group, A(H3N2) was the most identified subtype (75.00%), followed by A(H1N1)pdm09 (24.87%), A(H7N9) (0.10%), and A(untype) (0.04%). Those proportions were correspondent to that in the SARI group-A(H3N2) (73.49%), A(H1N1)pdm09 (25.58%), A(H7N9) (0.93%), and A(untype) (0.00%) with *p*-value 0.067 (Table 1). 

### 3.4. Influenza Virus Detection Rate across Age, Sex and Season among Sampled ILI and SARI Patients

**Figure 3:**
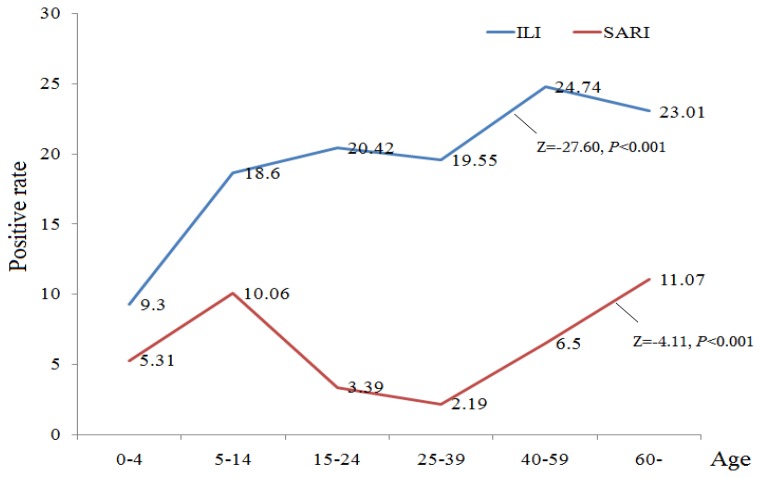
The influenza virus detection rate among the sampled ILI and SARI cases by age group in Zhejiang Province, China, March 2011–June 2015 (ILI = Influenza-like illness; SAIR = severe acute respiratory illness).

Influenza viruses were found in the specimen of 8601 of 52,293 (16.44%) all patients, with 8260 of 46,868 (17.62%) ILI patients and 341 of 5425 (6.29%) SARI patients. Table 2 shows the specific positive rate of influenza virus by age groups, genders, and seasons. For ILI patients, the highest (24.74%) rate is in the 40–59 years age-group, followed by >60 years age-group (23.01%). Meanwhile, the positive rate of influenza viruses of SARI patients was highest in the >60 years age-group (11.07%), followed by 5–14 years age-group (10.06%). Among both ILI and SARI cases, influenza virus was found in all age groups, and Cochran-Armitage trend test showed that the influenza virus positive rates tend to be higher at older ages (Figure 3). The positive rate was highest in the winter, and lowest in the autumn. Overall, the influenza virus positive rate among ILI cases was significantly higher than that among SARI cases across different groups of age, sex and season (Table 2).

### 3.5. Seasonality and Circulation of Influenza Types and Subtypes among ILI and SARI Cases

**Figure 4:**
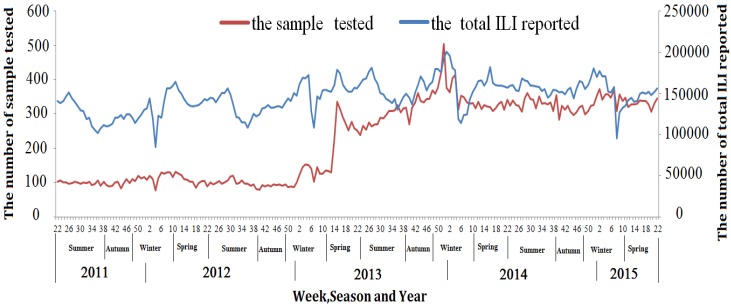
Temporal distribution of the weekly number of the samples tested and all ILI patients, in Zhejiang Province, March 2011–June 2015. (ILI = Influenza-like illness).

Due to the outbreak of avian H7N9 virus in April 2013, ILI surveillance was strengthened by increasing the number of samples for testing from 5–15 to 20. Figure 4 shows that although the weekly number of the samples tested has increased since the week 14 of year 2013, the weekly number of all ILI patients remained at similar level from year 2011 to 2014 (Figure 4).

**Figure 5:**
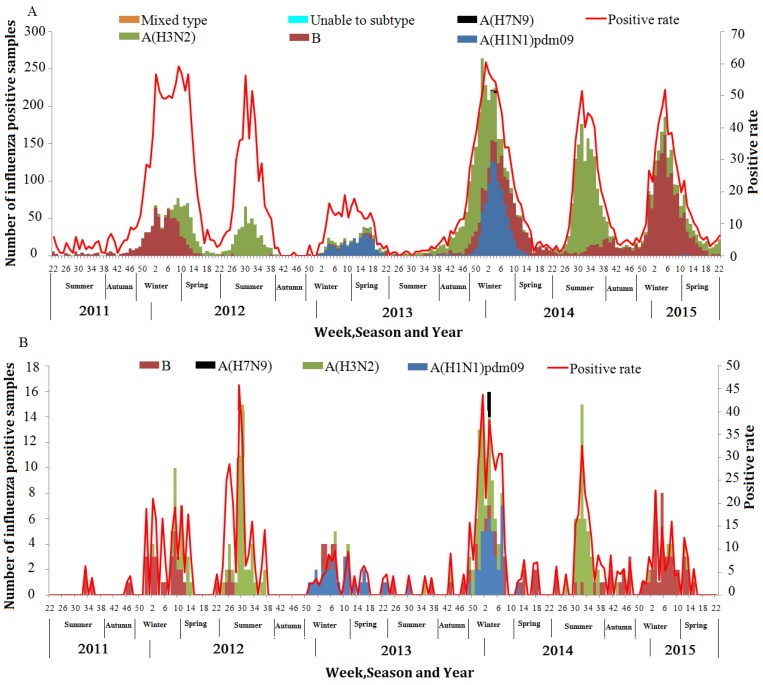
Temporal distribution of influenza virus-positive specimens and the percentage of specimens testing positive, in Zhejiang Province, March 2011–June 2015. (**A**) Influenza-like illness (ILI) influenza virus-positive patients (N = 8260); (**B**) Severe acute respiratory illness (SARI) influenza virus-positive patients (N = 341).

The seasonal patterns of influenza virus were shown in Figure 5. The winter-spring peaks were observed in each of the study year for ILI, and the summer peaks were only observed in the year of 2012 and 2014. 

The strengthened surveillance after the A(H7N9) outbreak led to the great increase of total number of flu positive samples from 2011–2012 to 2013–2015 (Figure 5A), while the positive rate at peaks remained largely the same in the whole study period, except for the 2012/2013 winter, which may be caused by the abnormal weather condition (the higher humidity and lower temperature), the emerging A(H7N9) virus and the vaccination campaign for A(H1N1)pdm09 launched in previous years. A(H3N2) was the predominant types/subtypes of the summer peaks in 2012 and 2014, while the predominant types/subtypes of the winter-spring were different across study years; influenza B is predominant and co-circulated with A(H3N2) during the 2011–2012 winter-spring peak; A(H1N1)pdm09 during the 2012–2013 winter-spring; a mix of A(H3N2), influenza B, and A(H1N1)pdm09 during the 2013–2014 winter-spring; and influenza B during the 2014–2015 winter-spring (Figure 5A). Consistently, this influenza activity was also observed among SARI patients (Figure 5B). Seven A(H7N9) viruses (five in the ILI group and two in the SARI group) were detected during the early year of 2014 (Figure 5). We found that the weekly percentage of influenza virus types/subtypes among all the identified influenza cases were significantly correlated between SARI and ILI patients, with A(H1N1)pdm09 (r = 0.51, *p* < 0.001), influenza B virus (r = 0.17, *p* = 0.013), and A(H3N2) (r = 0.43, *p* < 0.001) (Table 3).

### 3.6. Correlation Analysis between Weekly ILI Influenza-Positive Rate and SARI Influenza-Positive Rate, ILI Percentage and ILI Influenza-Positive Rate, SARI Percentage and SARI Influenza-Positive Rate

Table 4 shows the results of Spearman correlation analysis among influenza virus positive rate and percentage of people with ILI or SARI. The positive rate for influenza virus of ILI cases and those of SARI cases was statistically significantly correlated (r = 0.63, *p* < 0.001). The percentage of ILI cases and ILI influenza-positive rate was statistically significantly correlated (r = 0.53, *p* < 0.001), which was higher than it of SARI (r = 0.19), whose coefficient was statistical significant though (*p* < 0.001).

## 4. Discussion

To our knowledge, this is the first study to compare the epidemic characteristics of influenza between outpatients and hospitalized inpatients in Zhejiang Province. Children less than 5 years of age were found to be the largest group of both ILI and SARI patients, which was consistent with those reported by other studies [10,16,17]. Besides, we found a good agreement between SARI and ILI patients for the weekly proportion of samples tested positively for influenza virus and the distribution of the influenza virus types/subtypes among all the identified patients. This demonstrated that the seasonal pattern and predominant circulation types of influenza were similar between ILI and SARI patients. Finally, we found that the correlation between the weekly influenza positive rates and percentages of patients meeting the definition of ILI and SARI is higher among ILI patients than it among SARI patients, which indicated that compared to ILI, influenza may less important in causing SARI.

Although the largest age groups were same (0–4 years old) among ILI patients and SARI patients, this age group had higher proportion within SARI patients than it within ILI patients. This difference might be caused by the different behaviors when a child or an adult is found to be sick. Compared to adults, children are more likely to be taken to hospital, especially for SARI cases. Therefore, children have SARI were more likely to be involved in the surveillance. Similar results have been obtained in Mongolia [10], Philippines [11], Jordan [23]. Our findings further demonstrated that young children are vulnerable for both mild and severe respiratory infection, and the low influenza detection rate among 0–4 years age-group in both SARI and ILI patients foreshadow the need of expand the respiratory illness surveillance to more types of pathogens [12,24].

Being consistent with other studies, influenza virus was detected in all age groups among both ILI and SARI cases [10,14]. And overall, the proportion of samples tested positively for influenza viruses in different age groups presented to be higher with the increase of age. Therefore, all persons aged 6 months and older are recommended for vaccination and elders should be considered with priority [25].

To understand the temporal characteristics of influenza epidemics is essential for planning influenza vaccination programmes because vaccine effectiveness wanes over time, a boost of vaccine is essential to prevent the spread of diseases [26]. The simple and preferred measure to assess and compare seasonality patterns was the proportion of influenza positives [27]. The highly correlated influenza virus positive rate between SARI and ILI patients demonstrated the similar temporal pattern of influenza activity in the two groups. Similar findings were recently reported in a description of influenza surveillance in Egypt, which showed that the seasonality of influenza among ILI cases and SARI cases was consisted in November–February [19]. Moreover, the comparison between ILI patients and SARI patients shows no statistically significantly difference in detecting influenza virus type and influenza A virus subtype, which was similar to the findings from Nigeria [16]. Finally, the correlation of weekly percentage of influenza virus type/subtypes accounted for all the positive numbers between SARI and ILI patients indicated that the predominant influenza types/subtypes among ILI and SARI cases was corresponded. These findings are essential for planning influenza vaccination programmes for those severe cases given recommendations for strain inclusion within the vaccine are based on the ILI surveillance system. Our results were consistent to the study conducted by Peng et al. in the year of 2015 [4]. But in that study, the detailed analyses such as correlation analysis between weekly ILI influenza-positive rate and SARI influenza-positive rate, ILI percentage and ILI influenza-positive rate, SARI percentage and SARI influenza-positive rate were not performed. Therefore, we believe the present study can provide more comprehensive information on the comparison of influenza epidemiological and virological characteristics between outpatients and inpatients.

In line with the findings of other studies [11,13,14], we also found higher influenza detection rate among ILI patients compared to that among SARI patients. Moreover, we also found the correlation coefficient of ILI percentage and ILI influenza-positive rate was higher than that of SARI percentage and SARI influenza-positive rate. These phenomena may due to the higher specificity of the ILI diagnosis compared to the SARI diagnosis [28]. Some studies have demonstrated that influenza played an important role in the viral aetiologies of ILI cases [29,30,31], while other respiratory virus such as respiratory syncytial virus, rhinovirus, human bocavirus were essential for the cause of SARI, especially in children [32,33]. This indicated that it is more necessary to conduct pathogen spectrum test for SARI cases so as to accurately understand the cause of those severe cases.

Of note, we detected seven cases of A(H7N9) viruses (five in the ILI group and two in the SARI group) during early year of 2014, when this virus was outbreak in Zhejiang Province [34]. The detection rate of A(H7N9) virus in the SARI patients was higher than that in the ILI patients because this strain frequently cause severe syndromes. However, our study found that the surveillance network has low sensitivity on the capture of patients infected with A(H7N9) virus. Although one of the critical functions of influenza surveillance is to detect novel strains of influenza, the rapid detection of emerging novel influenza strains or outbreaks of respiratory disease calls for other surveillance with standardized methodology [5].

This study has several limitations. First, although SARI surveillance was required to catch all patients in accordance with the definition, sometimes the cases may not be fully recorded because of physicians’ oversights and absenteeism. In the ILI surveillance, to get all ILI patients surveyed was impossible due to the limited amount of resources and personnel. Therefore, the subject chosen for ILI sampled may prone to sicker patients or younger patients. Second, compared to ILI surveillance, SARI surveillance may be less representative due to its sparse surveillance sites. In the future, we should consider to expand and to enhance the coverage of the surveillance network, with priority to choose hospitals from ILI surveillance. Third, we did not test for pathogens other than influenza, which made us unable to exclude other viral, bacterial, and fungal pathogens that could be the causes of ILI and SARI.

## 5. Conclusions

This study demonstrated circulating types/subtypes of influenza strains and seasonality pattern of ILI cases were similar to that of SARI cases in Zhejiang Providence. This reassured the effectiveness of influenza vaccine as strain selection based upon ILI surveillance. Our study results suggest that compared to ILI patients, it is more necessary to conduct pathogen spectrum detection among SARI patients. In the future, the expanded and enhanced ILI and SARI surveillance in the province may contribute to identify the novel virus, detect pandemics at early stage, and then improve the understanding of the etiology of ILI and SARI.

## Figures and Tables

**Table 1:** Demographic and virologic characteristics of the sampled and tested patients by the influenza sentinel surveillance system of Zhejiang Province, during March 2011–June 2015.

Characteristic	Overall (%)	ILI (%)	SARI (%)	*p*-Value
Tested specimens	52,293	46,868	5425	
Age, year				
Median, IQR	15 (3–34)	18 (4–35)	1 (0.5–6)	<0.001
0–4	16,919 (32.35)	13,038 (27.82)	3881 (71.54)	
5–14	8318 (15.91)	7817 (16.68)	501 (9.24)	
15–24	5330 (10.19)	5284 (11.27)	46 (0.85)	
25–39	9516 (18.20)	9379 (20.01)	137 (2.53)	
40–59	6709 (12.83)	6355 (13.56)	354 (6.53)	
≥60	5501 (10.52)	4995 (10.66)	506 (9.33)	
Sex				<0.001
Male	27,070 (51.77)	23,747 (50.67)	3323 (61.25)	
Female	25,223 (48.23)	23,121 (49.33)	2102 (38.75)	
Seasons				<0.001
Spring	12,057 (23.06)	10,998 (23.47)	1059 (19.52)	
Summer	15,820 (30.25)	14,250 (30.40)	1570 (28.94)	
Autumn	9292 (17.77)	8348 (17.81)	944 (17.40)	
Winter	15,124 (28.92)	13,272 (28.32)	1852 (34.14)	
Influenza virus type detected				0.774
Influenza A virus	5334 (62.02)	5119 (61.97)	215 (63.05)	
Influenza B virus	3258 (37.88)	3132 (37.92)	126 (36.95)	
Mixed type	9 (0.10)	9 (0.11)	0 (0.00)	
Influenza A virus subtype detected				0.067
A(H3N2)	3997 (74.93)	3839 (75.00)	158 (73.49)	
A(H1N1)pdm09	1328 (24.90)	1273 (24.87)	55 (25.58)	
A(H7N9)	7 (0.13)	5 (0.10)	2 (0.93)	
A(Untype)	2 (0.04)	2 (0.04)	0 (0.00)	

IQR, interquartile range; ILI, influenza-like illness; SARI, severe acute respiratory illness.

**Table 2:** Number (and rate) of samples tested positively for influenza virus type among influenza-like illness (ILI) and severe acute respiratory illness (SARI) cases, during March 2011–June 2015.

Characteristic	ILI	SARI	*p*-Value
Overall	8260 (17.62)	341 (6.29)	0.001
Age, year			
0–4	1212 (9.30)	204 (5.31)	0.001
5–14	1543 (18.60)	51 (10.06)	0.001
15–24	1228 (20.42)	2 (3.39)	0.001
25–39	1834 (19.55)	3 (2.19)	0.001
40–59	1572 (24.74)	23 (6.50)	0.001
≥60	871 (23.01)	58 (11.07)	0.001
Sex			
Male	4017 (16.92)	220 (6.62)	0.001
Female	4243 (18.35)	121 (5.76)	0.001
Seasons			
Spring	1135 (10.32)	38 (3.59)	0.001
Summer	1951 (13.69)	106 (6.75)	0.001
Autumn	614 (7.36)	15 (1.59)	0.001
Winter	4560 (34.36)	182 (9.83)	0.001

ILI, influenza-like illness; SARI, severe acute respiratory illness. The *p*-value is for the comparison of the influenza positivity between ILI and SARI patients across each stratum.

**Table 3:** Correlation analysis of weekly influenza virus type/subtype constitution among total positive numbers between influenza-like illness (ILI) and severe acute respiratory illness (SARI).

Variable	Correlation Coefficient	*p*-Value
A(H1N1)pdm09	0.51	<0.001
A(H3N2)	0.43	<0.001
Influenza B	0.17	0.013

**Table 4:** Correlation analysis between weekly ILI influenza-positive rate and SARI influenza-positive rate, ILI percentage and ILI influenza-positive rate, SARI percentage and SARI influenza-positive rate.

Variable	Correlation Coefficient	*p*-Value
ILI influenza-positive rate and SARI influenza-positive rate	0.63	<0.001
ILI percentage and ILI influenza-positive rate	0.53	<0.001
SARI percentage and SARI influenza-positive rate	0.19	0.006
